# Anterior Approach to Suprascapular Nerve Block Provides Effective Analgesia for Shoulder Pain Following Thoracic Surgery

**DOI:** 10.5152/TJAR.2023.21502

**Published:** 2023-02-01

**Authors:** Mete Manici, Belitsu Salgın, Yavuz Gürkan

**Affiliations:** Department of Anaesthesiology and Reanimation, Koç University Hospital, İstanbul, Turkey

Dear editor,

Postoperative ipsilateral shoulder pain (PO-ISP) is common following thoracic surgery. Postoperative ipsilateral shoulder pain results from various challenges: referred pain of phrenic nerve, shoulder ligament strains, and patient positioning. Since PO-ISP is fairly resistant to intravenous analgesics, various regional analgesia methods have been tried, ranging with varying success rates.^1^ Some regional anaesthesia techniques include erector spinae plane block or interscalene block.

Suprascapular nerve block (SSNB) is widely used and can alleviate PO-ISP in thoracotomy patients up to 24-48 hours. A new anterior approach to SSNB (ASSNB) is introduced by Siegenthaler et al.^2^ Since ASSNB blocks more nerve branches and provides analgesia for a wider anatomic space, it can be clinically more effective than posterior approach.^3^ The articular branch of the suprascapular nerve (medial subacromial branch) emerges proximal to suprascapular notch; therefore, in theory, only anterior approach can successfully block this branch.^4^

A 63-year-old American Society of Anesthesiologists (ASA) II woman underwent a 90-minute thoracoscopic wedge resection under general anaesthesia. Induction was done with 2-3 mg kg^-1^ propofol, 1 mg kg^-1^ fentanyl, and 0.6 mg kg^-1^ rocuronium. Depth of anaesthesia was maintained with 1 MAC desflurane and 0.02-0.2 µg kg^-1^ min^-1^ remifentanil infusion. Patient received paracetamol 1 g IV, tramadol 100 mg IV, and ibuprofen 400 mg IV, intraoperatively, as part of multimodal analgesia. Thoracic epidural was inserted at T6 level. The epidural had bupivacaine with a background infusion rate of 6 mg h^−1^ and a bolus dose of 6 mg with 15 minutes of lock period. Although patient had no pain in thoracic region, severe shoulder pain with a numeric rating scale (NRS) of 8/10 in the first postoperative hour was present. The pain did not respond to the rescue analgesic, 30 mg meperidine, applied twice. Anterior approach to SSNB was performed as described by Siegenthaler et al.^2^ After supine positioning, the ultrasound high-frequency linear probe (8-18 MHz) of GE Logiq S7 (General Electric Healthcare, Little Chalfont, United Kingdom) was placed transversely on the ipsilateral neck where brachial plexus was visualized. The probe was moved towards the supraclavicular region, simultaneously while visualizing the suprascapular nerve. A 50-mm, 22-G regional block needle (BBraun, Melsungen, Germany) was introduced in-plane, lateral to medially, and 5 mL of 0.25% bupivacaine (5% Marcaine®, AstraZeneca PLC, United Kingdom) was injected perineurally under ultrasound guidance (Figure 1). Patient reported NRS pain score of 2/10, 30 minutes after the block, and 1/10, at the end of first hour. The patient was followed up for 24 hours and reported a NRS pain score of 2-3/10 in the 3rd, 6th, 12th, and 24th postoperative hours. Since it is a selective nerve block, low doses of local anaesthetic can be sufficient for postoperative analgesia. A systematic review and meta-analyses of SSNB revealed a range of 7-15 mL of 0.2%-1.0% ropivacaine use for the SSNB.^5^ We think that 5 mL of 0.25% bupivacaine can be effective.

To the best of our knowledge, this is the first case showing effective use of ASSNB for PO-ISP. We think ASSNB may become a safe and alternative analgesia method for PO-ISP. However, future comparative studies with larger patient populations are needed.

## Figures and Tables

**Figure 1.**
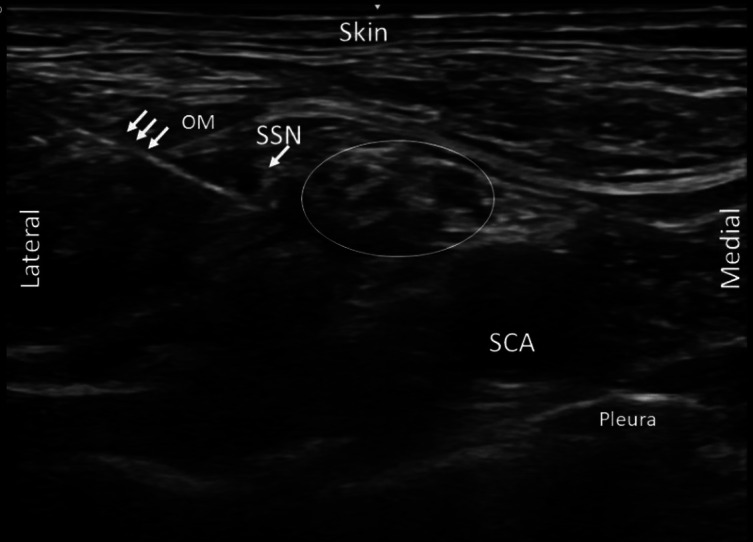
Ultrasonographic image of the supraclavicular fossa with the transducer positioned in an oblique sagittal plane. OM, omohyoid muscle (triple arrow); SCA, subclavian artery; SSN, suprascapular nerve (single arrow); needle and encircled is the brachial plexus.
